# Hoarding Behaviour in Autism Spectrum Disorders: A Deep Study of Two Cases

**DOI:** 10.1192/j.eurpsy.2026.11098

**Published:** 2026-07-31

**Authors:** U. Altunoez, T. Joedicke, A. Ellafi, A. Al Hamidi, C. Schwanke

**Affiliations:** General Psychiatry & Psychotherapy, KRH Wunstorf Psychiatric Hospital, Wunstorf, Germany

## Abstract

**Introduction:**

Hoarding behavior is defined as difficulty discarding belongings regardless of value, leading to cluttered living spaces and functional impairment. Although often seen in autism spectrum disorders (ASD), further research is needed to clarify the relationship between these phenomena.

**Objectives:**

This presentation aims to evaluate the descriptive and phenomenological aspects of hoarding in ASD and to explore—through two cases—whether it represents a distinct phenomenon or a manifestation of autism-related restrictive behaviors.

**Methods:**

Two cases from a psychiatric and psychotherapeutic training clinic in Lower Saxony, Germany, are presented.

**Results:**

**Case 1**

A 50-year-old male was referred by his legal guardian after remaining housebound for two years in a cluttered apartment. His mother, with whom he had lived, died two years earlier. He reported no long-term relationships and described social interaction difficulties. Intellectual level was normal; no psychotic symptoms were present apart from behavioral disorganization due to social withdrawal.

He had trained as a publishing clerk but lost his job due to social difficulties and has been unemployed for 15 years. Evaluation revealed autistic traits: social impairments, rigid routines, and restricted interests. Autism Quotient score was 41, supporting high-functioning ASD. Developmental history and clinical interview confirmed the diagnosis. He was unable to discard newspapers, other items, his personal hygiene had deteriorated, particularly since the death of his mother.

**Case 2**

A 57-year-old male was referred by his sister after his mother’s death two months earlier. He had stopped eating and drinking adequately and had become homebound. Social difficulties had been present since childhood. After high school, he trained as a motor mechanic but stopped working due to social challenges and perceived bullying.

He had long hoarded bottles and yogurt pots at home, a behavior that worsened after his mother’s death, with whom he had lived for many years. Developmentally autistic traits were evident, clinical evaluation supported high-functioning ASD.

Brain MRI was normal in both patients.

**Intervention and Outcome**

A systemic approach was used, including social skills training and supportive therapy for grief. In both cases, social difficulties and hoarding worsened after maternal loss, as their mothers had been both attachment figures and central to daily routines. Psychoeducation about ASD opened new perspectives, and phenomenologically, hoarding was understood as part of restrictive behaviors rather than a separate disorder.

**Conclusions:**

Hoarding behavior in ASD must be evaluated case-specifically. In many patients, it may represent routine-driven behavior that can intensify after life events, such as the loss of a family member who provided stability and attachment. Psychoeducation and systemic therapeutic approaches with social skills training are crucial in treatment.

**Disclosure of Interest:**

None Declared

